# Potential Ecological Risk Index and Metal Fate in a Karstic Tropical Lagoon: Chelem, Yucatan, Mexico

**DOI:** 10.1007/s00128-025-04156-0

**Published:** 2025-12-09

**Authors:** F. Arcega-Cabrera, K. León-Aguirre, E. Lamas-Cosío, J. A. Martínez-Trejo, I. Oceguera-Vargas

**Affiliations:** 1 Unidad de Química en Sisal, Facultad de Química, Universidad Nacional Autónoma de México, Puerto de Abrigo S/N , 97356 Sisal, Yucatán México; 2 Escuela Nacional de Estudios Superiores, Universidad Nacional Autónoma de México, Tablaje 6998, Carr. Mérida-Tetiz Km 4.5, 97357 Ucú, Yucatán México

**Keywords:** Contamination, Groundwater, Karstic lagoon, Potential damage to biota, Sediments

## Abstract

Chelem is a karstic lagoon with urban settlements, economic activities, and environmental services, making it highly vulnerable to metal contamination. This study evaluated metal sediment contamination using enrichment factor, geoaccumulation index and ecological risk index across dry, rainy and Nortes seasons. Cadmium and arsenic concentrations exceed the SQuiRTs thresholds for early biota damage. Metal distribution was driven by submerged groundwater discharges, co-transport with fine particular matter in rainy and Nortes seasons, and direct inputs from urban wastewater and port operations. Cadmium, arsenic and tin exhibited minor to moderate enriched levels. Geoaccumulation index classified the lagoon from minor to highly contaminated. Despite this, the overall ecological risk index remained low. However, sediment-bound metals may already affect local biota and pose future risks to fisheries and human health. These findings highlight the need for continued monitoring and improved management to prevent long-terms ecological and public health impacts in this vulnerable karstic system.

**Supplementary Information:**

The online version contains supplementary material available at 10.1007/s00128-025-04156-0.

## Introduction

Coastal lagoons are especially vulnerable to man driven physical and chemical changes (Barragán and Borja 2012) related with land use changes and inappropriate waste management. For centuries humans have lived and profited from the ecosystemic services provided by coastal areas, from food to materials and leisure activities (Mehvar et al. 2018). Nowadays, around 40% of the world population inhabits within 100 km to the coast (Ávila-Foucat and Espejel 2020), however, socioeconomic development in these areas is often unsustainable, promotes environmental degradation (López-Pacheco et al. 2019) and significantly affects coastal systems (Figueroa-Zavala et al. 2015).

Yucatan is a karstic platform where groundwater works as the means of transport for inland contaminants (Pérez-Ceballos et al. 2012, 2022; Arcega-Cabrera et al. 2021). The karst aquifer system is known for its high permeability and porosity, which stems from its intricate network of caves, fissures, and underground rivers (Perry et al. 1995, 2002; Guo et al. 2025), this geological structure allows for rapid groundwater discharge through these conduits and fractures (Perry et al. 1995, 2002). Groundwater containing contaminants is generally diluted by flowing recharge waters, however, the presence of contaminants could influence the interaction between chemical reactions of the hydrochemistry of the aquifer, like the dissolution of carbonate rocks (limestone and dolomite) (Shevenell and McCarthy 2002; Guo et al. 2025).

In the Yucatan Peninsula since only 4% of the wastewater is treated, likewise, in situ sources commonly use inefficient septic tanks and lack proper waste management strategies (urban and industrial). Therefore, it is probable that metals are being released into the coastal areas and are probably being accumulated in the sediments of coastal lagoons.

In addition, the hydrodynamics in a coastal lagoon could promote the storage of anthropogenic contaminants in the sediments (Arcega-Cabrera et al. 2009, 2014, 2015) which could present a risk to the benthic communities, those contaminants may also be released to the water column under tropical storms, hurricanes, etc., thereby exposing the pelagic biota. The monitoring of contaminants, like metals, in sediments could indicate the environmental quality, particularly where fish are consumed by humans, and therefore work as an early alert for human health risk (Castañeda-Chávez et al. 2017, Robledo-Ardila et al. 2023).

To evaluate the potential ecological risk; Wang et al. (2015) developed a series of geochemical indexes that allow a comparison in the overtime increase of metals in sediments and contributes to the development of mitigation strategies (Devanesan et al. 2020).

Fishing is one of the main activities in these coastal lagoons, therefore, it is of great importance to determine the potential contamination and environmental risk to identify if metal contents in sediments could be of concern for the socioenvironmental system and especially human health. Insufficient wastewater management in Chelem lagoon could led to the accumulation of trace metals in sediments, posing potential ecological and human health risks, this study aims to determine metal distribution in sediments of Chelem lagoon and evaluate their ecological risk using geochemical indexes (Igeo), the enrichment factor (EF) and the potential ecological risk index (ER), to evaluate the probable ecological risk.

## Materials and Methods

### Study Area

**Fig. 1:**
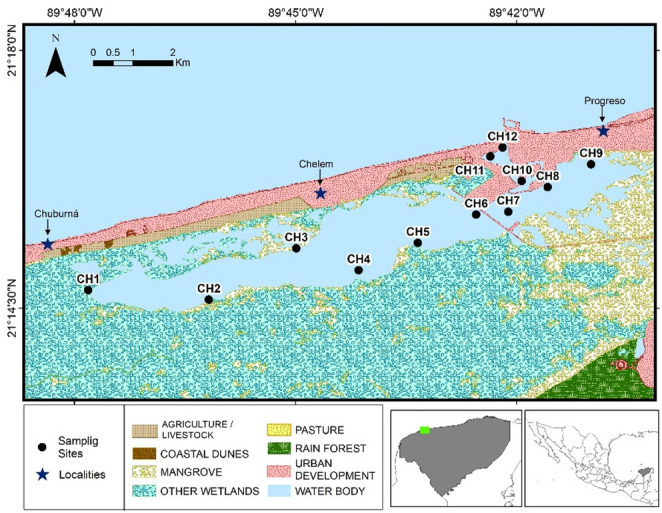
Sampling sites and land use in Chelem lagoon, Yucatan, Mexico

Chelem lagoon is located in the north side of the Yucatan península between 21° 15´ to 21° 17´N and 89° 39´ to 89° 48´W. Chelem is part of the natural protected area named “Ciénegas y manglares de la costa norte de Yucatán”. It has an area of 13.6 km^2^ that varies significantly through the year with the floding-dissication rates related to the main three hydrological seasons, dry, rainy and north, and also the occurence of hurricanes or tropical storms. It holds the port of Yucalpetén, where there is a great lagoon-ocean interaction (Tenorio Fernández 2015). Salinity gradient has been reported by Valdés et al. (1992), with values from 65 ups in dry season to 10 ups in north season, as a result of the high evaporation in dry season and the heavy precipitation and increased volume of submarine groundwater discharges in rainy and north seasons, but also wastewater (urban and industrial) direct inputs (Arcega-Cabrera et al. 2014). Main activities are port related, fishing, tourism and urban settlements occupy a large extension (Fig. 1).

## Sampling and Metal Analysis

Surface sediments (ca. 1–3 cm depth) from selected sites in Chelem (Fig. 1) were collected (we use a composite sample composed of three subsamples collected one meter apart and subsequently homogenized in a single container), refrigerated, freeze dried and sieved (500 mm mesh) following the methods of Loring and Rantala (1992) and Arcega-Cabrera et al. (2014) as well as microwave digested using the Environmental Protection Agency method (EPA 3052 MF 100-T16). Metals were analyzed using an Atomic Absorption Espectophotometer Annalyst 800 coupled with air/acethylene flame, graphite furnace and hidride generator. Analytical quality assurance was obtained by using a SRM 1646a Estuarine Sediment. Mean percentage recovery was, 97.93, 90.50 and 98.51% from As, Cd and Sn respectively. The detection limit was 0.0013 µg/g 0.0020 µg/g and 0.0003 mg/g from As, Cd and Sn respectively. For the rest of the metals please refer to Table 3 at supplementary material.

## Geochemical Indexes

These indexes were obtained according to Wang et al. (2015) as follows.

Enrichment Factor (FE) is used to differentiate natural and anthropogenic metals. M_sed_ = metal concentration and M_c_ = metal concentration in the crust.

$$\:FE=\frac{\left(\frac{{M}_{sed}}{{Al}_{sed}}\right)}{\left(\frac{{M}_{c}}{{Al}_{c}}\right)}$$

Geoacumulation index (Igeo) is the metal pollution index where C_n_ is the metal concentration in sediments and B_n_ is the metal background concentration.

$$\:{I}_{geo}={log}_{2}\left(\frac{{C}_{n}}{{1.5B}_{n}}\right)$$

Ecological risk potential, evaluates the probable risk from metal pollution, were i = metal, Tri is the biological toxicity factor for a given element (i), Cf, Cs and Cn = contamination factor, metal concentration in sediments and the background value respectively, and IR = is the sum of Rei for several metals (Hakanson 1980).

$$\:{RE}_{f}^{i}={Tr}^{i}{xC}_{f}^{i}={Tr}^{i}x\left(\frac{{C}_{s}^{i}}{{C}_{n}^{i}}\right)\:IR=\sum\:_{f}^{i}{RE}_{f}^{i}$$

### Statistical Analysis

Prior to hypothesis testing, the data were explored for distributional properties and homogeneity of variances. Normality was examined with the Shapiro–Wilk test at a 95% confidence level, and variance homogeneity was evaluated with Levene’s test. Since most datasets did not conform to the assumptions of parametric statistics, the non-parametric Kruskal–Wallis test was applied to detect significant differences in both spatial (among sampling sites) and temporal (among seasons) scales. When the Kruskal–Wallis test indicated significant variation (*p* < 0.05), post hoc pairwise comparisons were carried out using Dunn’s test with Bonferroni adjustment to identify specific groups responsible for differences. All analyses and graphical outputs were performed using OriginLab Pro 2019, which allowed robust visualization and statistical interpretation of the results.

### Mapping

Geospatial analysis was conducted using ArcMap 10.8 software. The spatial distribution of each index was represented using graduated color symbology based on natural breaks (Jenks) classification, which minimizes intra-class variance and maximizes inter-class variance. This method allows a more accurate visualization of spatial gradients and hotspots across the study area. In cases where concentrations were below the detection limit (< LD), the detection limit value was substituted during index calculation to avoid data gaps and maintain consistency in the spatial interpolation. These values were incorporated into the mapping process, ensuring that all sites were represented. Additionally, interpolation techniques (Inverse Distance Weighting, IDW) were tested to evaluate spatial continuity, although only classified symbology was retained in the final maps to avoid overinterpretation of point data. The mapping procedure thus provides a reliable visualization of the spatial patterns, supporting the statistical analysis.

## Results

Metal mean concentration in dry, rainy and north seasons is shown in Table 1 (supplementary material). Concentrations of As and Cd exceeded the thresholds established in the SQuiRTs (Screening Quick Reference Tables; Buchman 2008) in all seasons, indicating a potential toxicological risk to early stages of aquatic biota. These metals are likely introduced through both local sources—such as untreated wastewater, poultry-related discharges, and open waste burning (Castro-González et al. 2019; Arcega-Cabrera and Fargher 2016)—and regional processes associated with submarine groundwater discharge (SGD). In karst environments like Yucatán, alkaline pH conditions favor As mobilization (Sun et al. 2023), and the presence of interconnected sinkholes accelerates contaminant transport to the coastal system (Metcalfe et al. 2011). Intensive and widespread agrochemical application also contributes to Cd and As presence in groundwater and coastal sediments (González et al. 2008; López-Pacheco et al. 2019). Agrochemicals is a serious contamination problem on the Yucatan coast due to the porosity and permeability and agricultural practices. The porous limestone bedrock allows pollutants to easily leach into the aquifer, which is the primary source of drinking water (González et al. 2008). High concentrations of Cd and As represent damage to early stage of biota (Marques Quintanela et al. 2020, Reyes Marquez et al. 2024), as well as a health risk by consumption of commercial biota contaminated with this heavy metals (Reyes Marquez et al. 2024).

Regarding metal distribution, the spatial differences (Table 2 supplementary material) were statistically significant for Ni, Zn and Sn. Ni and Sn showed higher concentrations in the dry season indicating an input from localized anthropogenic sources, like urban and industrial wastewater and significant concentrations of Sn from the antifouling paints used for boat maintenance (Fujita et al. 2015; Telegdi et al. 2016). Zn showed high concentrations during the rainy season, suggesting a potential contribution from SGD or runoff processes (Arcega-Cabrera et al. 2014; Kantún-Manzano et al. 2018).

Significant seasonal differences (Table 2 supplementary material) were found for Al, Mn, As and Sn, indicating a temporal trend, with higher values during the dry season. This pattern can be attributed to reduced hydrological activity during dry season (Murray Tortarolo 2021), which enhances sedimentation of particles and concentrates contaminants due to limited dilution and flushing (Metcalfe et al. 2011), also higher sedimentation of metals co-transported with suspended material in this season, given the low flow and the absence of strong winds, tropical storms or hurricanes (Arcega-Cabrera et al. 2014). Under these conditions, local sources are likely to have a greater influence than SGD, particularly where residence time of pollutants is elevated.

**Fig. 2:**
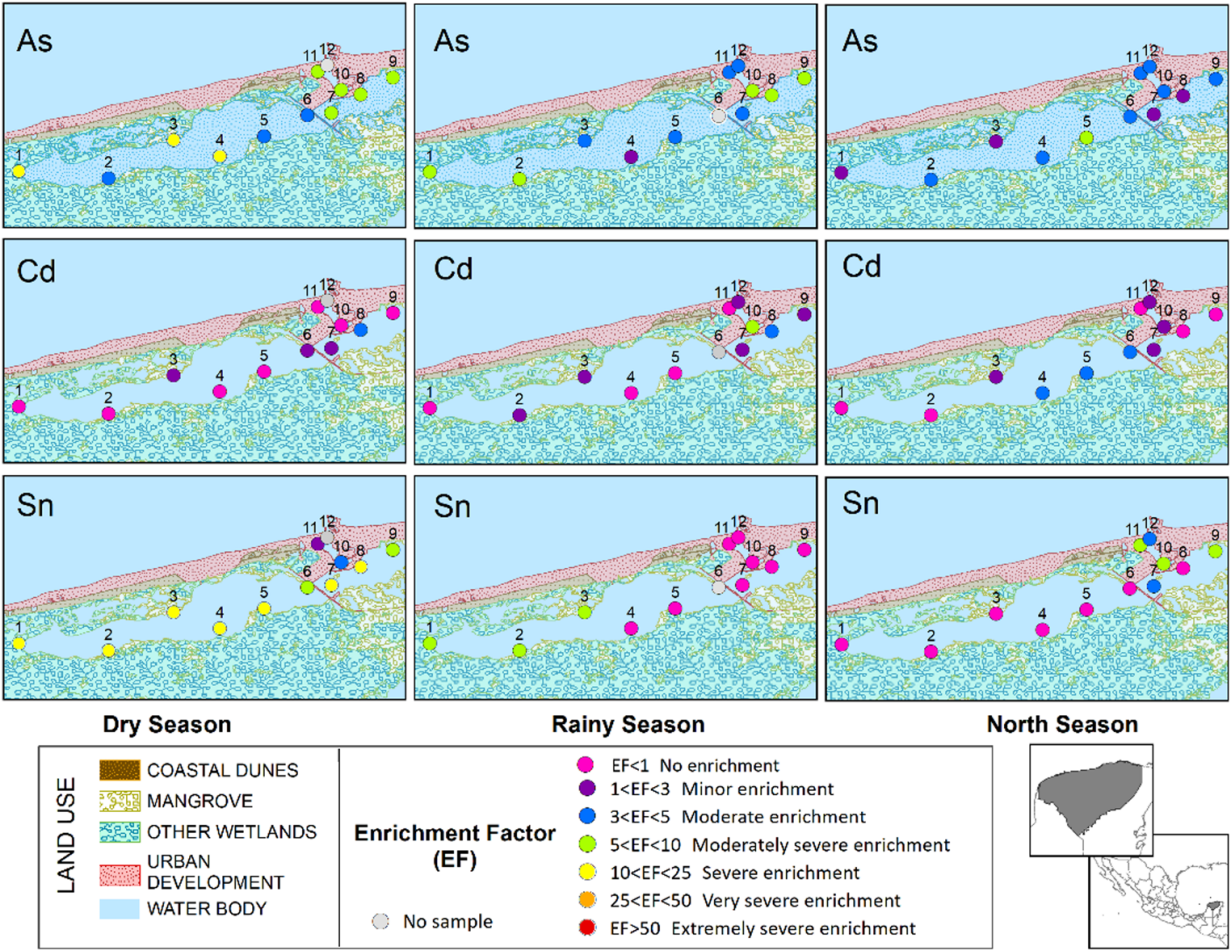
Map of distribution of values of Enrichment factor (EF) categorization

The enrichment factor (EF) analysis (Fig. 2; Table 4 Supplementary Material) indicated minor to severe enrichment for Cd, As, and Sn, confirming sustained anthropogenic input to the lagoon. Cd high levels in groundwater have been previously reported (Pacheco-Ávila et al. 2004), but the concentration in the SGD has not been measured, therefore further research in this SGD outlet should be done for all elements to confirm that local inputs are greater than SGD ones. The highest values of EF for As and Sn were observed during the dry season and across multiple sites, supporting the hypothesis of local source contributions combined with sediment co-transport and deposition (Arcega-Cabrera and Fargher 2016; Sun et al. 2023). The presence of Sn is directly linked to port activities, while As enrichment may reflect both aquifer inputs and direct discharge, facilitated by the physicochemical conditions of the sediment-water interface (Perry et al. 2002).

But the fact that for these elements the higher concentrations are found in dry season and all over the lagoon, could be confirming that local inputs and distribution with suspended matter could be the process guiding As and Sn fate. In the case of Sn, as mentioned before, the port in Chelem has a high activity regarding boats maintenance using antifouling paint, and the wastewater of this industry is poured directly into the lagoon.

**Fig. 3:**
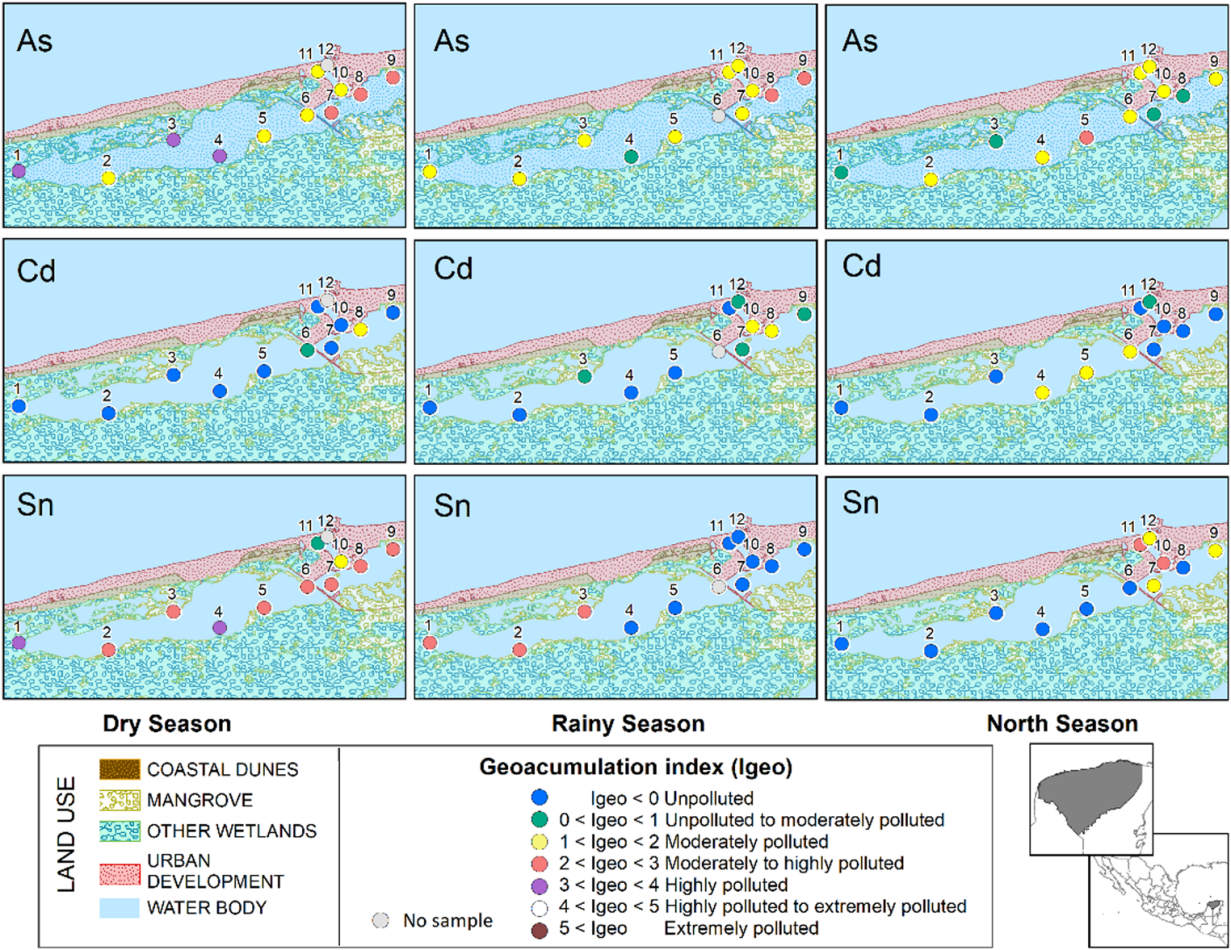
Geoaccumulation index for sediments of Celem lagoon, Yucatan, Mexico

Geoaccumulation index (Igeo) values (Fig. 3; Table 5 Supplementary Material) showed non- to moderate contamination for Cd and up to high contamination for As and Sn. These results are consistent with EF values and highlight both the intensity and spatial extent of contamination by these elements. The overlap in spatial and seasonal trends between Igeo and EF further supports the role of local contamination sources.

Ecological risk index (RI) values remained within the low-risk category for all analyzed elements, including Cd, despite its elevated concentration relative to toxicity thresholds. This result may be associated with sediment properties such as grain size, organic matter content, and pH, which can influence the mobility and bioavailability of metals (Hamid et al. 2022). However, this does not preclude bioaccumulation and trophic transfer, particularly in sediment-associated fauna such as bivalves or benthic invertebrates. These organisms may act as exposure vectors for higher trophic levels, including fish and birds, and represent a potential route of human exposure.

The concentrations and geochemical behavior Cd, As, and Sn observed in Chelem lagoon sediments are consistent with contamination patterns reported in other karstic environments, like the chinese karstic system (Dinis et al. 2021; Yu et al. 2022; Lian et al. 2024). In Chelem, Cd presented enrichment factors ranging from minor to severe, and geoaccumulation indices classified sediments as non to moderately contaminated, particularly during the dry season. Comparable results were reported in karst lake systems in China, where Cd also exhibited moderate to high geoaccumulation values and ecological risk classifications, despite spatial variability in hydrological and anthropogenic inputs (Dinis et al. 2021). The high mobility of Cd in alkaline karst environments, coupled with low natural attenuation capacity, has been emphasized as a key driver of sediment enrichment in both soils and lacustrine systems (Yu et al. 2022; Lian et al. 2024). Similarly, the chronic enrichment of As observed in Chelem aligns with findings from subtropical karst basins, where mobilization from agricultural and domestic sources is enhanced by carbonate-rich conditions (El-Sharkawy et al. 2025). In contrast to non-karstic systems, where As may be sequestered by clays or organic matter, karst terrains exhibit high As bioavailability due to reduced sorption capacity. Sn, although less frequently studied in karst regions, showed enrichment and contamination levels in Chelem lagoon like those detected in other semi-enclosed systems with high boating activity, reinforcing the role of local antifouling practices as dominant sources. Additionally, studies of subterranean estuarine communities in karst regions have demonstrated that fluctuations in precipitation and SGD can modulate contaminant exposure pathways and biological responses (Calderón-Gutiérrez et al. 2023), suggesting that the seasonal patterns observed in Chelem may reflect a broader vulnerability of karstic coastal systems to hydrologically driven contaminant dynamics.

Nevertheless, the enrichment and contamination levels of the studied metals are of concern, Cd is known to impair enzymatic function, reproduction, and growth in benthic invertebrates and fish, even at low concentrations, and can accumulate in edible tissues, thereby entering human food chains (El-Sharkawy et al. 2025; Zeng et al. 2024). Similarly, As is highly toxic and can interfere with cellular respiration and DNA repair mechanisms in marine biota. In karstic systems, the high mobility of As increases its availability in sediments and facilitates its uptake by detritivores and filter feeders (El-Sharkawy et al. 2025). Sn, commonly introduced via tributyltin (TBT) and other organotin compounds from antifouling paints, is known to disrupt endocrine function, causing reproductive anomalies such as imposex in gastropods and impairing larval development in crustaceans (Collado et al. 2010; Zeng et al. 2024). Evidence shows that chronic exposure to Cd and related metals in sediments correlates with reduced biodiversity and altered benthic community structure (Alharbi et al. 2025). These effects are particularly relevant in shallow, semi-enclosed coastal lagoons such as Chelem, where sediment-bound metals can be remobilized by hydrometeorological events, increasing exposure risk to pelagic species and further propagating contamination through ecological networks.

## Conclusions

Chelem lagoon exhibits moderate to high levels of sediment contamination by As, Cd, and Sn, based on concentration data, enrichment factors, and geoaccumulation indices. These metals are introduced primarily by local anthropogenic activities such as wastewater discharge, boat maintenance, and agrochemical use, with a secondary contribution from submarine groundwater discharge.

Seasonal patterns indicate higher metal concentrations during the dry season, corresponding to conditions of low hydrological exchange and enhanced sedimentation. This supports the predominance of local sources over SGD in contaminant input during this period.

Despite the classification of ecological risk as low, the persistence of metal accumulation in sediments, along with concentrations exceeding biological effect thresholds, suggests potential long-term impacts on benthic communities and trophic networks. Current conditions facilitate chronic exposure scenarios, which may lead to bioaccumulation and possible health risks for wildlife and humans.

Monitoring and mitigation strategies should prioritize the control of local inputs, including the implementation of wastewater treatment infrastructure and regulation of port activities. Future research should address the bioavailability and speciation of metals, as well as biomonitoring of exposed fauna, to better quantify ecological and human health risks in this karstic coastal system.

## Supplementary Information

Below is the link to the electronic supplementary material.

Supplementary Material 1
