# Supplementary material for: Potential Ecological Risk Index and Metal Fate in a Karstic Tropical Lagoon: Chelem, Yucatan, Mexico
Source: Bull Environ Contam Toxicol. 2025 Dec 9;116(1):4. doi: 10.1007/s00128-025-04156-0 (PMC12689742; doi:10.1007/s00128-025-04156-0)
Supplement: Supplementary file 1 — Supplementary Material 1 [file 128_2025_4156_MOESM1_ESM.docx]

Supplementary material

Table 1. Metal mean concentration, minimum and maximum values at each season in the composite sediment samples of Chelem lagoon.

| **Chelem** | | | | | | | | | | |
| --- | --- | --- | --- | --- | --- | --- | --- | --- | --- | --- |
| **Metal/Season** | | | | | | | | | | |
| **Al** | **Cr** | **Cd** | **Cu** | **Fe** | **Mn** | **Ni** | **Pb** | **Zn** | **As** | **Sn** |
| **(%)** | **(µg/g)** | **(µg/g)** | **(µg/g)** | **(µg/g)** | **(µg/g)** | **(µg/g)** | **(µg/g)** | **(µg/g)** | **(µg/g)** | **(mg/g)** |
| **dry** | | | | | | | | | | |
| 1.52 | 1.48 | 0.06 | 0.007 | 0.014 | 28.5 | 2.61 | 0.75 | 10.1 | 9.29 | 0.059 |
| 1.33-2.05 | 0.149-16.2 | 0.013-0.47 | 0.001-0.011 | 0.004-0.751 | 15.27-71.61 | 2.08-9.12 | 0.09-8.08 | 3.58-55.7 | 5.44-30.97 | 0.016-0.077 |
| **rainy** | | | | | | | | | | |
| 0.264 | 3.35 | 0.15 | 0.01 | 0.026 | 17.1 | 2.79 | 1.59 | 20.1 | 7.35 | 0.045 |
| 0.080-2.89 | 0.23-4.66 | 0.005-0.52 | 0.002-0.025 | 0.004-0.100 | 1.88-33.6 | 1.36-4.31 | 0.78-28.1 | 4.43-58.7 | 2.26-11.2 | 0.042-0.049 |
| **north** | | | | | | | | | | |
| 0.21 | 1.45 | 0.1 | 0.01 | 0.014 | 21.9 | 3.36 | 2.15 | 22.9 | 5.22 | 0.03 |
| 0.063-0.576 | 0.188-6.21 | 0.030-0.338 | 0.004-0.025 | 0.001-0.148 | 8.11-44.5 | 1.13-6.86 | 0.05-17.5 | 4.79-115 | 2.66-10.06 | 0.003-0.04 |

*Values in red are above the SQuiRTs (Buchman, 2008) values for potential damage to the early stages of the biota as follows: Cd=0.34, Cr=49, Cu=32, Pb=30, Ni=15, Zn=94, Fe=22000, Mn=260, As=7.4, Sn=0.5 and Al=800000

Table 2: Spatial and time significant differences in Chelem lagoon. Underlined values = significant differences.

| **Chelem** | | | | | | | | | | |
| --- | --- | --- | --- | --- | --- | --- | --- | --- | --- | --- |
| **Metal/p value** | | | | | | | | | | |
| **Al** | **Cr** | **Cd** | **Cu** | **Fe** | **Mn** | **Ni** | **Pb** | **Zn** | **As** | **Sn** |
| **Time** | | | | | | | | | | |
| <0.001 | 0.354 | 0.386 | 0.059 | 0.487 | 0.025 | 0.33 | 0.269 | 0.051 | 0.002 | 0.013 |
| **Spatial** | | | | | | | | | | |
| 0.936 | 0.41 | 0.219 | 0.086 | 0.235 | 0.149 | 0.005 | 0.459 | 0.014 | 0.29 | 0.039 |

Table 3. Recovery percentage and detection limit of the measured elements.

| Reference material  1646a | Al % | Cr µg/g | Cd µg/g | Cu µg/g | Fe % | Mn µg/g | Ni µg/g | Pb µg/g | Zn µg/g | As µg/g | Sn mg/g |
| --- | --- | --- | --- | --- | --- | --- | --- | --- | --- | --- | --- |
|  | 2.297 ± 0.018 | 40.9 ± 1.9 | 0.148 ± 0.007 | 10.01 ± 0.34 | 2.008 ± 0.039 | 234.5 ± 2.8 | 23.00 | 11.7 ± 1.2 | 48.9 ± 1.6 | 6.23 ± 0.21 | 1.00 |
| Recovery percentage | 97.05 | 93.49 | 90.56 | 94.66 | 89.62 | 87.54 | 90.48 | 88.65 | 92.25 | 97.94 | 98.51 |
| Detection limit | 0.0003 | 0.0107 | 0.0020 | 0.0003 | 0.0008 | 0.0005 | 0.0005 | 0.0003 | 0.0009 | 0.0013 | 0.0003 |

Table 4. Enrichment factor for metals content in sediments of the Chelem lagoon. Values categories are shown in the table below.


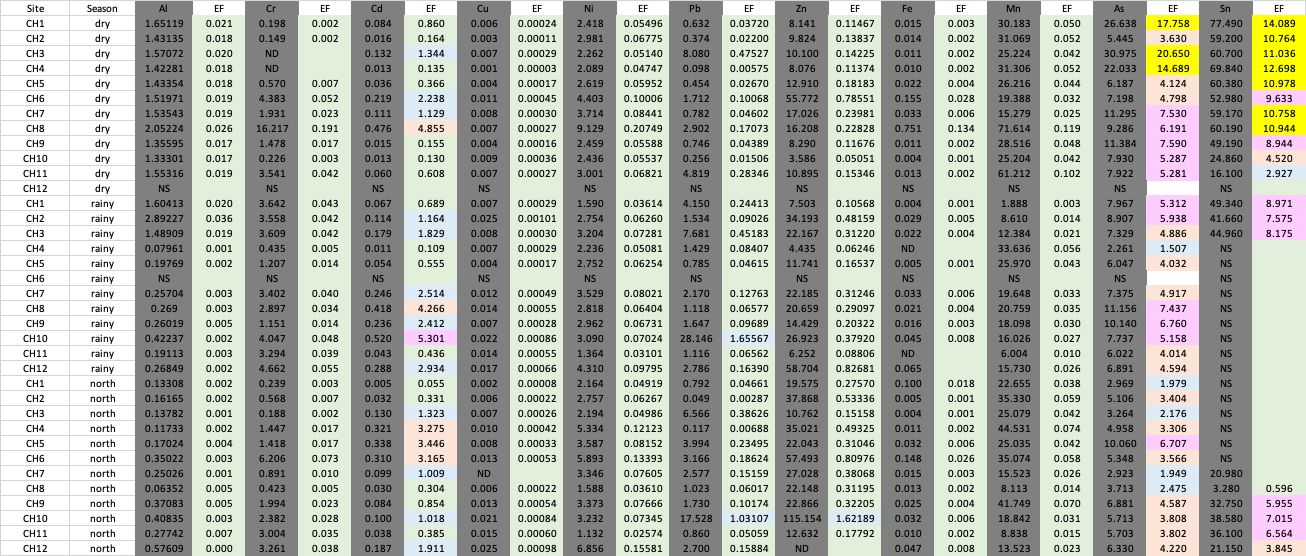


| **EF<1** | **No enrichment** |
| --- | --- |
| **1<EF<3** | **Minor enrichment** |
| **3<EF<5** | **Moderate enrichment** |
| **5<EF<10** | **Mildly sever enrichment** |
| **10<EF<25** | **Severe enrichment** |
| **25<EF<50** | **Highly sever enrichment** |
| **EF>50** | **Extremely severe enrichment** |

Table 5. Geoaccumulation index (Igeo) for metals content in sediments of the Chelem lagoon. Values categories are shown in the adjacent table. ND = non detected, NS = no sample.


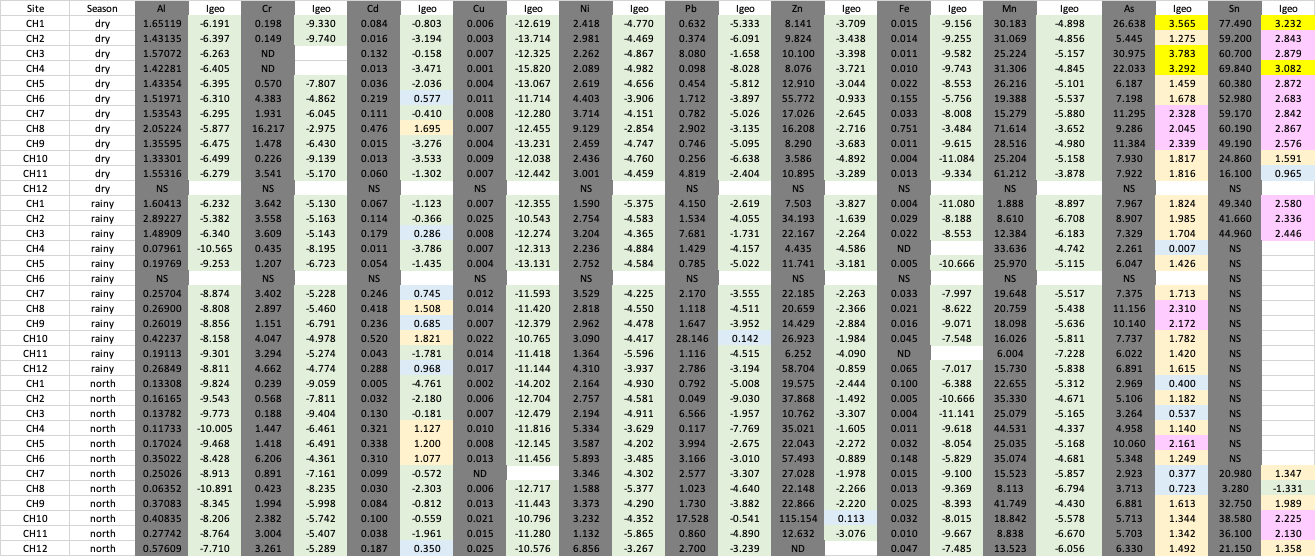


| Igeo<=0 | Class 0, non contaminated |
| --- | --- |
| 0<Igeo<=1 | Class 1, non to moderately contaminated |
| 1<Igeo<=2 | Class 2, moderately contaminated |
| 2<Igeo<=3 | Class 3, moderately to highly contaminated |
| 3<Igeo<=4 | Class 4, highly contaminated |
| 4<Igeo<=5 | Class 5, highly to extremely contaminated |
| Igeo>5 | Class 6, extremely contaminated |

Table 6. Potential ecological risk for metals content in sediments of the Chelem lagoon. Values categories are shown in the adjacent table. ND = non detected, NS = no sample.


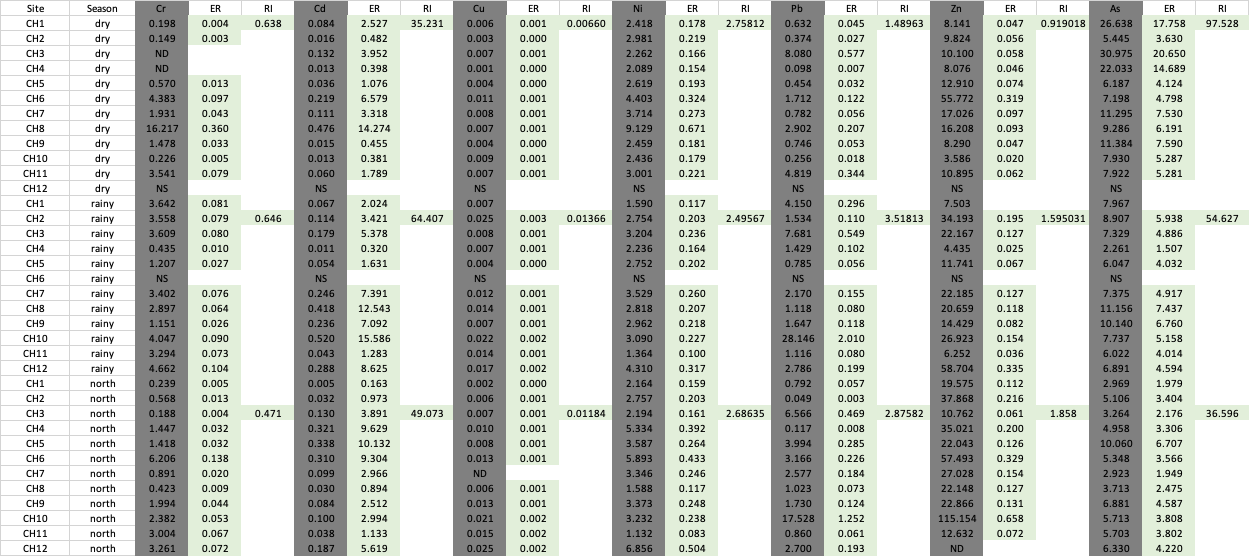


| **ER 6<=40 low risk** |
| --- |
| **40 < ER<80 moderate risk** |
| **80 < ER<160 considerable risk** |
| **160 <ER<320 high risk** |
| **ER >320 very high risk** |
| **RI<=150 low risk** |
| **150<RI<=300 moderate risk** |
| **300 <RI<=600 considerable risk** |
| **RI>600 very high risk** |
